# “Silicon-On-Insulator”-Based Biosensor for the Detection of MicroRNA Markers of Ovarian Cancer

**DOI:** 10.3390/mi14010070

**Published:** 2022-12-27

**Authors:** Yuri D. Ivanov, Svetlana I. Kapustina, Kristina A. Malsagova, Kristina V. Goldaeva, Tatyana O. Pleshakova, Rafael A. Galiullin, Ivan D. Shumov, Andrey F. Kozlov, Alexander V. Glukhov, Victoria K. Grabezhova, Vladimir P. Popov, Oleg F. Petrov, Vadim S. Ziborov, Nikolay E. Kushlinskii, Alexander A. Alferov, Vladimir A. Konev, Oleg B. Kovalev, Vasiliy F. Uchaikin, Alexander I. Archakov

**Affiliations:** 1 Institute of Biomedical Chemistry (IBMC), 119121 Moscow, Russia; 2 Joint Institute for High Temperatures of Russian Academy of Sciences, 125412 Moscow, Russia; 3 Department of Cybernetics of Chemical and Technological Processes, Mendeleev University of Chemical Technology of Russia (MUCTR), 125047 Moscow, Russia; 4 JSC “Novosibirsk Plant of Semiconductor Devices with OKB”, 630082 Novosibirsk, Russia; 5 JSC “Design Center for Biomicroelectronic Technologies “Vega””, 630082 Novosibirsk, Russia; 6 Rzhanov Institute of Semiconductor Physics, Siberian Branch of Russian Academy of Sciences, 630090 Novosibirsk, Russia; 7 N.N. Blokhin National Medical Research Center of Oncology, 115478 Moscow, Russia; 8 Department of Infectious Diseases in Children, Faculty of Pediatrics, Pirogov Russian National Research Medical University (RNRMU), 117997 Moscow, Russia

**Keywords:** ovarian cancer, miRNA, biomarkers, oDNA, silicon-on-insulator, nanowire, biosensor

## Abstract

Ovarian cancer is a gynecological cancer characterized by a high mortality rate and tumor heterogeneity. Its early detection and primary prophylaxis are difficult to perform. Detecting biomarkers for ovarian cancer plays a pivotal role in therapy effectiveness and affects patients’ survival. This study demonstrates the detection of microRNAs (miRNAs), which were reported to be associated with ovarian cancer tumorigenesis, with a nanowire biosensor based on silicon-on-insulator structures (SOI-NW biosensor). The advantages of the method proposed for miRNA detection using the SOI-NW biosensor are as follows: (1) no need for additional labeling or amplification reaction during sample preparation, and (2) real-time detection of target biomolecules. The detecting component of the biosensor is a chip with an array of 3 µm wide, 10 µm long silicon nanowires on its surface. The SOI-NW chip was fabricated using the “top-down” method, which is compatible with large-scale CMOS technology. Oligonucleotide probes (oDNA probes) carrying sequences complementary to the target miRNAs were covalently immobilized on the nanowire surface to ensure high-sensitivity biospecific sensing of the target biomolecules. The study involved two experimental series. Detection of model DNA oligonucleotides being synthetic analogs of the target miRNAs was carried out to assess the method’s sensitivity. The lowest concentration of the target oligonucleotides detectable in buffer solution was 1.1 × 10^−16^ M. In the second experimental series, detection of miRNAs (miRNA-21, miRNA-141, and miRNA-200a) isolated from blood plasma samples collected from patients having a verified diagnosis of ovarian cancer was performed. The results of our present study represent a step towards the development of novel highly sensitive diagnostic systems for the early revelation of ovarian cancer in women.

## 1. Introduction

Ovarian cancer is characterized by a high mortality rate and tumor heterogeneity: tumors differ in their clinicopathological and molecular features, thus hindering early diagnosis and primary prophylaxis of this disease. However, although there are many subtypes of ovarian cancer, all of them are considered to be the same disease [1,2]. As of 2022, the American Cancer Society estimated that ovarian cancer ranks fifth in cancer deaths among women in the USA. Ovarian cancer is responsible for more deaths than any other cancer of the female reproductive system. It is most likely to develop in older women: approximately 50% of all female patients having a verified diagnosis of ovarian cancer are older than 63 years [3,4]. The five-year survival rate does not exceed 70% and varies depending on the pathology subtype.

Ovarian cancer is often diagnosed only at later stages. This circumstance is the main cause of the high mortality rate. The latter is the reason why early diagnosis is necessary. The currently available screening methods are, however, far from being perfect. The screening tests for ovarian cancer include pelvic examination, transvaginal ultrasound, computed tomography or magnetic resonance imaging of the pelvis and abdomen, ovarian biopsy, and posterior fornix biopsy [5]. In addition, because of the intraperitoneal location of the ovaries, surgical intervention is required for the diagnosis. It is not recommended to use needle biopsy in early ovarian cancer, since this procedure may induce cancer spread into the abdominal cavity [6]. The subtle symptoms responsible for the late detection of this disease, as well as the lack of adequate screening, contribute to poor diagnosis and ineffective treatment [7].

Detection of specific biomarkers, which are expected to allow for sufficiently accurate and rapid diagnosis, is currently used for early diagnosis of ovarian cancer. These biomarkers include carbohydrate antigen 125 (CA 125), which was recommended by the Food and Drug Administration (FDA). However, despite the CA 125 test that is commonly used in healthcare practices, early diagnosis of ovarian cancer still remains a challenge. In addition, CA 125 tests often provide false-positive results due to the lack of specificity [8]. Enzyme linked immunosorbent assay (ELISA), which allows one to attain a concentration detection limit of ~10^−12^ M, is employed for detecting protein biomarkers of ovarian cancer, such as CA 125 [9]. In this regard, it is to be emphasized that in the case of ovarian cancer, the specificity of differential diagnosis of malignant tumors using the CA 125 marker is low, since CA 125 is associated with inflammation, while not being produced by cancer cells. This determines the high relevance of the search for novel specific biomarkers of ovarian cancer.

MicroRNAs (miRNAs) were recognized as suitable markers for several types of human cancer, including ovarian cancer [6,10]. MiRNAs represent short (approximately 18–25 nucleotide long) non-coding RNA sequences, which can regulate the expression of several target genes in normal biological processes such as cell cycle control, proliferation, differentiation, and apoptosis [11]. MiRNAs are stable in various bodily fluids such as blood, saliva, and urine. Functional experiments have confirmed the oncogenic or tumor suppressor effects of several miRNAs [6]. Increased expression of several miRNAs, including miRNA-21, miRNA-141, miRNA-200a, miRNA-221, miRNA-718, miRNA-506, etc., in patients with ovarian cancer was also reported [6,12]. In the literature, miRNAs are increasingly often mentioned as biomarkers of ovarian cancer [6,13]. Samples for miRNA detection can be easily obtained by non-invasive methods, since they circulate in bodily fluids such as blood, urine, and saliva.

To date, the conventional miRNA detection methods include Northern blotting, sequencing, isothermal exponential amplification, microarray analysis, and quantitative polymerase chain reaction (qPCR) [14,15]. The detection limit attainable by qPCR is typically ~10 DNA copies per reaction in 100 µL [16]. The drawbacks of these conventional methods are as follows: the complexity of data analysis, sample contamination with different nucleic acids that results in false positive or false negative results, high reagent cost, and multistep protocols [15,16,17,18,19,20]. Accordingly, the development of highly sensitive methods of miRNA detection is required for the determination of low blood concentrations of these biomarkers. Furthermore, the developed technology should be selective and meet the requirements posed by the minimal sample volume, cost effectiveness, and the capabilities of a multiplexed assay [21].

The methods employing highly sensitive biosensors, which allow one to detect biomarkers in bodily fluids at low concentrations (<10^−13^ M) at early stages of pathology, can be considered to be promising [22]. One of these methods is based on the use of optical surface plasmon resonance (SPR) biosensors [23,24,25,26,27,28], including long-range SPR [29,30]. While the sensitivity of the long-range SPR is similar to that of conventional SPR, it allows one to obtain a signal from the thicker layer of the analyte solution. Long-range SPR allows one to investigate processes of intermolecular interactions in the frequency range of up to THz, despite the fact that its sensitivity does not significantly exceed that of conventional SPR [30]. Biosensors are sensitive and selective analytical devices consisting of two key components: a biological recognition (sensor) element for detecting the target miRNA, and a transducer element capable of converting the signal, resulting from interaction between the target biomolecule in the analyzed sample with the probe biomolecule immobilized on the sensor surface, to a measurable signal [31]. Table 1 lists the miRNA detection limits attainable with different types of biosensors intended for the detection of miRNAs associated with ovarian cancer. In comparison with conventional nucleic acid-based detection methods, biosensors display superior performance in terms of sample preparation, assay time, cost, portability, and complexity [14].

Promising next-generation biosensors include those based on nanostructures such as nanowires [36,37]. Nanowire biosensors based on silicon-on-insulator (SOI) structures occupy a special place: they are manufactured using the CMOS-compatible top-down approach [38,39], which is eventually intended to be used for large-scale production. In the present study, this very technology has been employed for the fabrication of a SOI-NW sensor chip, which was based on n-type field-effect transistors. In our previous papers, we demonstrated that biosensors of this type are characterized by high sensitivity for detecting influenza A virions, protein molecules, and circRNA [40,41,42,43]. This system allows one to conduct real-time analysis without using any additional labels, and has high sensitivity, allowing one to attain the detection limit of 10^−16^ to 10^−15^ M. 

In comparison with other types of biological macromolecules, miRNAs have a number of advantages, which make them ideal candidate biomarkers of various diseases. An ideal biomarker should be easily extractable, and miRNAs are just the case: one can easily extract these macromolecules from blood, urine, and other bodily fluids by liquid biopsy [44]. In comparison with protein biomarkers, miRNAs are much less complex. In addition, miRNAs are stable in various bodily fluids. With regard to various clinically relevant diseases, nucleotide sequences of many miRNA biomarkers are highly conserved. MiRNAs have high specificity to the tissue type. The levels of marker miRNAs depend on the disease stage [45]. To date, technologies of miRNA detection are available, and the development of novel methods of miRNA detection requires less time and less resources—as compared to obtaining new antibodies against protein biomarkers. MiRNAs can also be used as multi-marker models for accurate diagnosis and estimation of therapy effectiveness, while the use of many protein markers can be expensive and laborious [44]. As regards viral particles as biomarkers [40,46], their disadvantage consists of the need for preliminary inactivation, which can be detrimental for the sample integrity at the molecular level [47,48]. 

In the present study, a SOI-NW biosensor was used for the detection of three types of miRNA (miRNA-21, miRNA-141, and miRNA-200a) isolated from plasma, which are associated with ovarian cancer, as suggested in the literature [6,21]. To provide biospecific interaction analysis, the silicon nanowire surface was sensitized by the covalent immobilization of oDNA probes. These oDNA probes represent oligonucleotides carrying sequences complementary to the regions of miRNA sequences. Five blood plasma samples of women with a confirmed ovarian cancer were analyzed. It was found that the SOI-NW biosensor allows one to detect signal intensity reduction for the samples collected from the patients with ovarian cancer—in comparison with the control samples of patients with diagnosed breast cancer and the samples of healthy volunteers.

## 2. Materials and Methods

### 2.1. Reagents

The following chemicals were used in this study to chemically modify the surface of the the SOI-NW chip: isopropanol purified to 99.9% (C_3_H_8_O) (Acros Organics, Geel, Belgium), ethanol (C_2_H_5_OH, 96%) (Reakhim, Moscow, Russia), and hydrofluoric acid (HF) (Reakhim, Moscow, Russia). The sensor surface of the SOI-NW chip was activated using a 3,3′-dithiobis(sulfosuccinimidyl propionate) (DTSSP) crosslinking agent (Thermo Fischer Scientific, Waltham, MA, USA). Potassium phosphate monobasic buffer (PPMB) KH_2_PO_4_ (Sigma Aldrich, St. Louis, MO, USA) was used as the buffer solution. Silanization was carried out using 3-aminopropyltriethoxysilane (APTES) (Sigma-Aldrich, St. Louis, MO, USA). The deionized water used in this study was purified on a Simplicity UV system (Millipore, Molsheim, France).

### 2.2. Oligonucleotides

All the oligonucleotides used in this study were synthesized by Evrogen (Moscow, Russia). The oDNA probes denoted as probe_1, probe_2, and probe_3 were employed for sensitizing the nanowire surface. Their sequences are listed in Table 2. Table 3 summarizes the sequences of model DNA oligonucleotides, the synthetic analogues of respective miRNAs. These probes were designated as CS and used as oDNA targets in a series of experiments to determine the method’s sensitivity. The CS oligonucleotide sequence is complementary to oDNA probes with the respective numbering.

The oDNA probe specific to the breast cancer marker [49] (5′-(NH_2_)-(T)_10_-CACGACCGACGCCACGCCG, designated as probe_4) and the oDNA probe not specific to any cancer marker (5′-(NH_2_)-T_10_ CATGGTTGCCACATG, designated as probe_5) were used to take into account the non-specific signal. The oDNA probes probe_4 and probe_5 were immobilized on the surface of nanowires, which were chosen as controls in the experiment.

**Table 3:** Nucleotide sequences of oDNA targets and miRNAs corresponding to them.

oDNA	The Sequence of oDNA (CS) Complementary to the oDNA Probe	Respective miRNA [Reference]
* CS 1	TGTCGGGTAGCTTATCAGACTGATGTTGACTGTTGAATCTCATGGCAACACCAGTCGATGGGCTGTCTGACA	hsa-mir-21 [10,50]
CS 2	CGGCCGGCCCTGGGTCCATCTTCCAGTACAGTGTTGGATGGTCTAATTGTGAAGCTCCTAACACTGTCTGGTAAAGATGGCTCCCGGGTGGGTTC	hsa-mir-141 [51,52]
CS 3	GCGGGTCACCTTTGAACATCGTTACCAGACAGTGTTAGAGTCAAGCTGGGAAATCCAGCACTGTCCGGTAAGATGCTCACAGGGGCCCGG	hsa-mir-200a [53]

* CS, Complementary Sequence.

**Figure 1:**
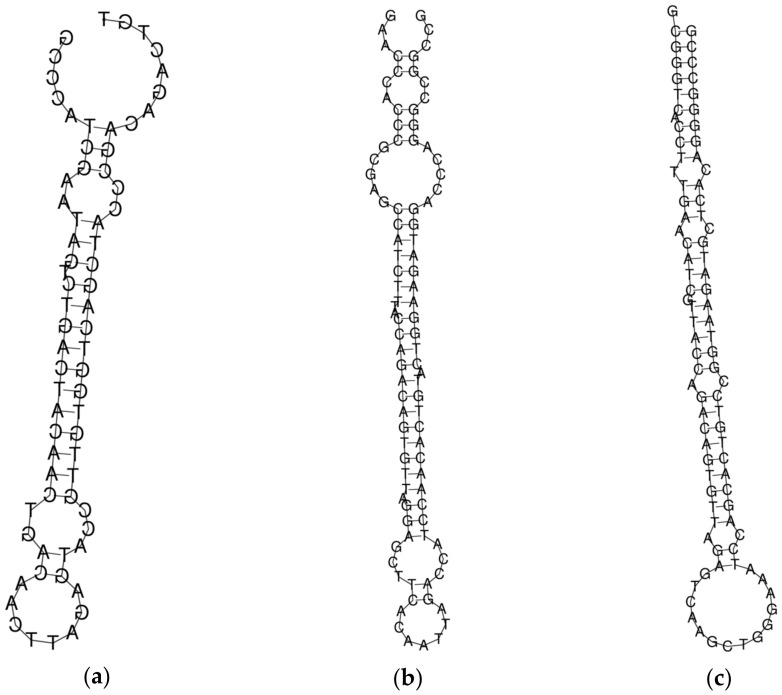
The structure of molecular probes probe_1 (**a**), probe_2 (**b**), and probe_3 (**c**) obtained using the RNAfold source (http://rna.tbi.univie.ac.at/cgi-bin/RNAWebSuite/RNAfold.cgi; accessed on 8 December 2022).

The structure of all the probes is shown in Figure 1. The free energy of the thermodynamic ensemble of probe_1, probe_2 and probe_3 is −21.11 kcal/mol, −37.58 kcal/mol and −44.18 kcal/mol, respectively. The minimum free energy was calculated using the RNAfold web server (http://rna.tbi.univie.ac.at/cgi-bin/RNAWebSuite/RNAfold.cgi, accessed on 8 December 2022).

### 2.3. Preparation of Solutions of oDNA Targets in Buffer

Solutions of oDNA targets (CS) with concentrations ranging from 10^−17^ M to 10^−14^ M were prepared from the initial solutions (100 μM in 50 mM PPMB, pH 7.4) by tenfold serial dilution in a buffer solution (1 mM PPMB, pH 7.4). At each dilution step, the solutions were shaken for 30 min at 10 °C. The oDNA solutions were prepared immediately before their use in measurements.

### 2.4. Patients’ Blood Plasma Samples

Blood samples used in the experiments were collected either from patients during medical examination or from healthy volunteers. Blood was collected from the ulnar vein of fasting patients into special vacuum tubes containing 3.8% C_6_H_5_O_7_•3Na anticoagulant (S-Monovette®, Sarstedt, Lower Saxony, Germany) and centrifuged at 3000 rpm for 6 min at room temperature. The blood samples were then separated into plasma and blood cells. The plasma samples were divided into 500 µL aliquots, which were then placed into sterile dry Eppendorf-type test tubes. Blood plasma samples were frozen at –80 °C and stored until their use in the experiments.

All the patients’ blood plasma samples were provided by the N.N. Blokhin National Cancer Research Center. Table 4 summarizes the characteristics of plasma samples used in the experiments. Plasma samples from patients with a verified diagnosis of ovarian cancer (No. 8, No. 10, No. 14, and No. 15) were examined. Plasma samples from healthy volunteers (No. 1 and No. 2) and a patient having a verified diagnosis of breast cancer (No. 80) were used in the control experiments. Experiments involving plasma were performed in compliance with the Order No. 1177n of the Russian Ministry of Healthcare dated 20 December 2012, and the verdict of an independent ethics committee. All the patients provided an informed consent for using their biological materials in this study. In order to provide biological safety, all the biomaterial was appropriately inactivated before being used in the experiments.

Total miRNA was isolated from blood plasma samples using the ExtractRNA reagent (Evrogen, Moscow, Russia) according to the protocol provided by Evrogen. This reagent is a monophasic aqueous solution of phenol and guanidine isothiocyanate and is used for rapid cell lysis. Blood plasma was mixed with the reagent, and further isolation was performed according to the manufacturer’s instructions immediately before the experiment. The isolated miRNAs were diluted with 75% ethanol [54].

### 2.5. Silicon-On-Insulator Nanowire Biosensor

**Figure 2:**
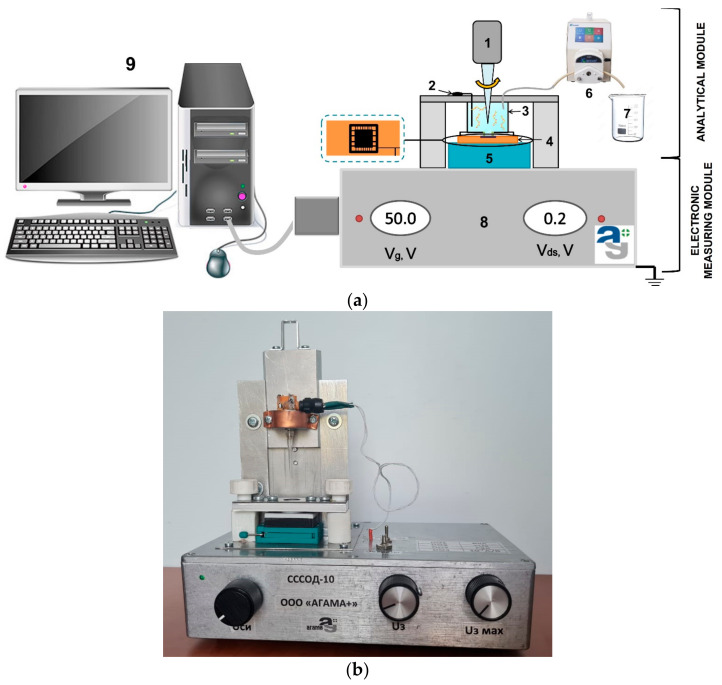
(**a**) A schematic view of the analytical module of a SOI-NW biosensor. Numbers indicate the key components of the module: (1) a stirrer; (2) a platinum electrode; (3) a measuring cell; (4) a SOI-NW chip; (5) a chip holder; (6) a peristaltic pump; (7) a waste container; (8) a ten-channel data collection and storage system, and (9) a PC. (**b**) A photographic image of the experimental setup.

The SOI-NW biosensor is a system consisting of two modules: the analytical and the electronic measuring modules (Figure 2). The analytical module consists of a 500 µL measuring cell, the surface of the SOI-NW chip being its bottom. The diameter of the sensitive area was ~2 mm. The solution in the cell was stirred at 3000 rpm with a stirrer. A platinum electrode was used to increase the stability of SOI-NW chips similar to [38,39].

**Figure 3:**
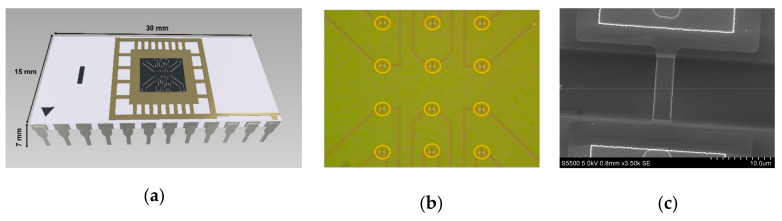
(**a**) The model of the SOI-NW chip being used. (**b**) An optical image of the surface of the SOI-NW chip. The arrangement of nanowires on the chip surface is outlined with orange color. (**c**) A SEM image of an individual silicon nanowire.

The key component of the analytical module is a chip bearing six pairs of silicon nanowires with n-type conductivity on its surface (Figure 3). The thickness of the cut-off silicon layer was 32 nm; the thickness of buried oxide (BOX) was 300 nm. Each nanowire was 3-µm-wide, 32-nm-thick, and 10-µm-long (Figure 3b,c).

The second module of the SOI-NW biosensor was the electronic measuring module, which simultaneously detected signals from ten nanowires located on a single chip and visualized them on the PC monitor during the experiment. The detected signals were transformed and visualized in a graphic form using specialized software (Agama+, Moscow, Russia).

**Figure 4:**
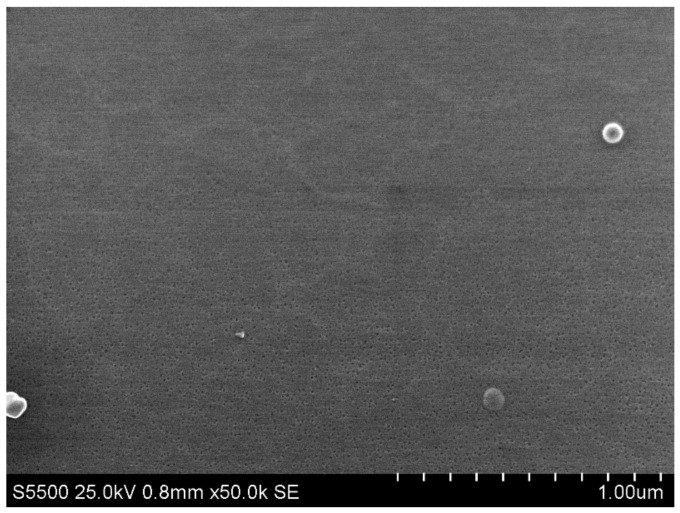
Scanning electron microscopy (SEM) image of the surface morphology of a silicon nanowire.

The chip was fabricated by top-down approach, and the fabrication steps were as follows: (1) fabrication of the initial silicon-on-insulator (SOI) structures, with a 500–600 nm thick cut-off Si layer using the hydrogen delamination technology; (2) reduction of the SOI layer to the nanoscale by a serial cycle of operations (thermal oxidation followed by oxide removal in HF solution); (3) lateral structuring of SOI layers using optical or electron beam lithography in order to form nanowire structures and Ohmic contacts on the SOI layer; (4) lateral structuring of SOI layers using electron beam lithography and gas–chemical plasma etching; (5) metal sputtering, contact wiring, and chip packaging. The manufacturing of SOI-NW chips was described in more detail in our earlier papers [38,39,41]. Figure 4 displays a SEM image showing the surface morphology of the silicon nanowire.

### 2.6. Chemical Modification and Sensitization of the Surface of the SOI-NW Chip

**Figure 5:**
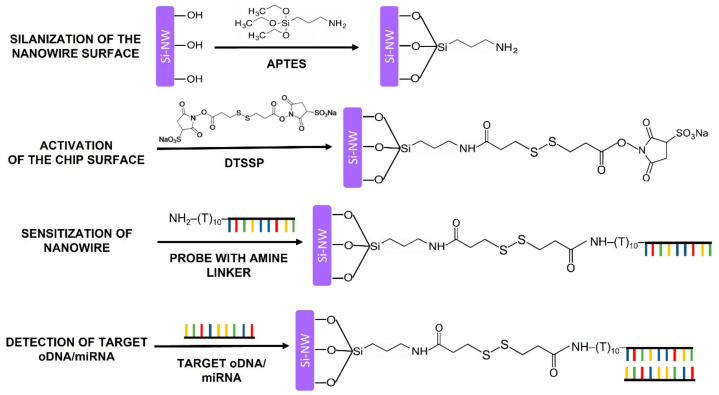
A schematic representation of the principle of the functionalization of the sensor surface. The functionalization comprised chemical modification and sensitization of the SOI-NW chip surface, which were performed in order to provide reliable detection of the target biomolecules.

Functionalization of the SOI-NW chip surface is required in order to provide its biological functionality by covalent immobilization of target-specific molecular probes on this surface. Proper surface functionalization provides high detection sensitivity and stable signal reproduction. The functionalization comprised two sequential stages: chemical modification and sensitization. The principle of the functionalization is schematically shown in Figure 5. Organic and mechanical contaminants were removed from the chip surface using an aqueous isopropanol solution during chemical modification. The SOI-NW chip surface was then treated with a mixture of hydrofluoric acid solution and ethanol to remove natural oxide, which had formed during the chip storage. After this treatment, the chip was placed into a UV Ozone Cleaner - ProCleaner™ Plus ozone generator (Ossila Ltd., Sheffield, UK) for 60 min so that hydroxyl groups were formed on the nanowire sensor surface. The nanowires were then silanized in APTES vapor in order to form a silane layer carrying terminal primary amine groups, which are required at the next functionalization stage according to a protocol similar to that reported elsewhere [38,39]. 

During the sensitization stage, the modified sensor surface of the SOI-NW chip was activated with a DTSSP crosslinker [38], which is a homobifunctional crosslinking agent commonly used for single-step covalent immobilization of biomolecules [55]. Esters mainly react with primary amine groups in biomolecules. In this study, a primary amine group was introduced into the oDNA probes during their synthesis (as shown in Table 2).

After the surface activation, the oDNA probes were sensitized by covalent immobilization of oDNA probes. For this purpose, 1 μM solutions of oDNA probes (from probe_1 to probe_5) in 50 mM PPMB (pH 7.4) were dispensed onto the activated surface of individual nanowires with high precision employing a non-contact iONE-600 robotic system equipped with a piezoelectric dispenser (PDMD) (M2-Automation GmbH, Berlin, Germany). The volume of the oDNA probe solution dispensed onto each nanowire was 1 nL. After the dispensing, the probe solutions were incubated on the nanowire surface for 30 min at 15 °C and 80% air humidity. After the immobilization stage, the chip surface was washed with deionized water.

Nanowires with immobilized oDNA probes (from probe_1 to probe_3), whose nucleotide sequences were complementary to miRNA-21, miRNA-141, miRNA-200a, and the respective oDNA targets were used as working sensors. In order to account for the non-specific signal, nanowires with immobilized oDNA probes complementary to miRNAs associated with breast cancer (probe_4) and the oDNA probe (probe_5) not complementary to any of the miRNAs used in this study were used as control sensors.

### 2.7. Electrical Measurements

Electrical measurements were carried out using the electronic measuring module, the 10-channel data collection and storage system designed at the Institute of Biomedical Chemistry in collaboration with Agama+ (Moscow, Russia). The current passing through the nanowire was digitized using an analog-to-digital converter and displayed on the PC monitor.

The NW-biosensor system allows measuring electrical signal in two modes: in the real-time mode and in the mode of recording the volt–ampere characteristics (VACs) of the nanowires. A VAC is the dependence of source-drain current *I_ds_* on applied gate voltage *V_g_*. The *I_ds_*(*V_g_*) dependence was recorded in buffer solution in the *V_g_* range from 0 to 60 V. After each functionalization step, chip performance was controlled by recording the VACs, which show the exponential dependence of nanowire current (conductivity) on surface potential. The working *V_g_* value was determined prior to the real-time biosensor measurements using the so-recorded VACs.

Dependence of source-drain current (*I_ds_*) on time (*t*) was recorded during real-time biosensor measurements described below. 

### 2.8. Conducting Experiments Using a SOI-NW Biosensor

All the experiments were carried out in a buffer solution with low salt concentration (1 mM potassium phosphate buffer) to reduce the Debye screening effect [56,57].

An SOI-NW chip with n-type conductivity, modified and sensitized with oDNA probes according to the procedure described in Section 2.6, was used in our studies. In the first series of experiments, biosensor-based detection of oDNA targets (CS 1, CS 2, and CS 3) in buffer solution was performed in order to determine the lowest concentration of oligonucleotides detectable with our SOI-NW biosensor. Taking into account the dilution of the sample upon its addition to the buffer solution in the measuring cell, the final concentration of the target oDNAs in the studied solutions ranged from 1.1 × 10^−17^ M to 1.1 × 10^−14^ M. The experiments were performed as follows: 150 μL of the analyzed solution of the CS target in 1 mM PPMB (pH 7.4) was added to the cell containing the SOI-NW biosensor and 300 μL of 1 mM PPMB. Three types of CS solutions were sequentially analyzed for each concentration, starting with the lowest one (10^−17^ M). After each analysis run, the measuring cell was washed with buffer solution and then with 50 mL of deionized water at 60 °C. In order to take into account the nonspecific signal, 1 mM PPMB containing no targets was added at the same ratios as those taken when analyzing CS solutions.

In the second series of experiments performed in order to detect miRNAs isolated from patients’ plasma samples, the following sequence of steps was used: 7 μL of a solution containing miRNAs isolated from plasma samples, collected from patients with confirmed ovarian cancer, was added to the measuring cell of the SOI-NW biosensor containing 100 μL of 1 mM PPMB (pH 7.4). The measurement protocol was similar to that used for the detection of various CS concentrations. Control experiments were performed under similar conditions, but a buffer solution containing miRNAs isolated from blood plasma samples, collected from a patient diagnosed with breast cancer and healthy volunteers, was added to the buffer the cell.

### 2.9. Data Analysis

The resulting data are presented as sensorgram curves: time dependencies of a dimensionless value expressed in relative units [40]. The detected signal intensity *I_ds_* for each nanowire was normalized by dividing its value by the initial current value. Next, in order to take into account the nonspecific interactions, the values obtained in the control experiment (i.e., using a buffer containing neither analyzed oDNA nor miRNA) were subtracted from the data obtained when analyzing the solution of the target oDNA or miRNA. The differential signal between the normalized signal from the working and control nanowires was then calculated. This data analysis allows one to account for the fact that different nanowires located on one and the same sensor chip have different initial current characteristics.

The conventional standard deviation function in Microsoft Excel was used to perform statistical analysis of the data obtained in the experiments.

## 3. Results

### 3.1. Controlling the Functionalization of the Nanowire Surface

**Figure 6:**
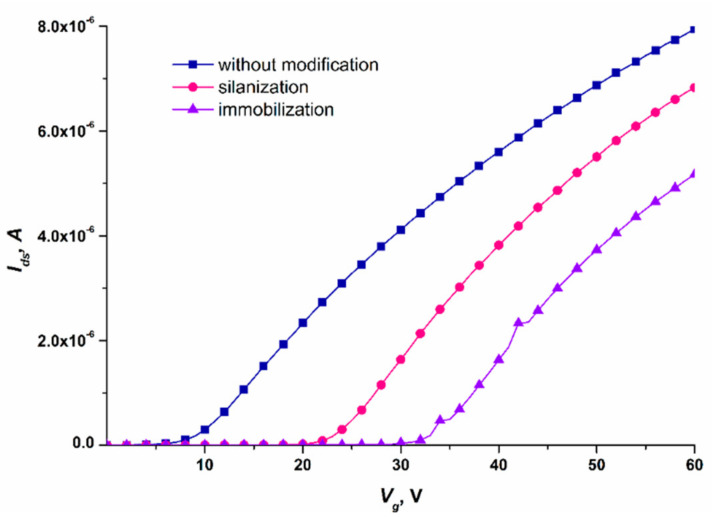
Typical volt–ampere characteristics recorded for one and the same nanowire on the SOI-NW chip with n-type conductivity. Measurement conditions: 1 mM PPMB; *V_g_* = 0 – 60 V, *V_ds_* = 0.2 V; the oDNA probe probe_1 was covalently immobilized on the nanowire. The VACs were recorded before the nanowire functionalization (blue curve), after the nanowire silanization (pink curve), and after its sensitization with oDNA probes (violet curve).

Before carrying out high-sensitivity biomolecule detection, we needed to test the performance of the SOI-NW chip after the functionalization and sensitization stages. For this purpose, comparative analysis of the volt–ampere characteristics (VACs) of nanowires with n-type conductivity, recorded before and after each stage of their functionalization, was performed. The working voltage for the real-time experiments was determined by analyzing VACs in the range from 0 to 60 V. Figure 6 displays typical VACs recorded for one and the same nanowire before its functionalization, and after each of the two functionalization stages.

The curves shown in Figure 6 indicate that after each functionalization stage, the *I_ds_*(*V_g_*) curve shifted to the right with respect to the curve recorded at the previous stage. Changes in the nanowire conductivity after chemical modification and sensitization of its surface were used for monitoring the efficiency of functionalization of the SOI-NW chip surface.

Having analyzed the VACs of the nanowires on the SOI-NW chip, the optimal working voltage parameters for the experiments were selected: *V_g_* = 50 V and *V_ds_* = 0.2 V. At lower gate voltage *V_g_*, the signal from the biosensor can drop to the minimum (or even to the background level), thus impeding the recording of the biosensor signal. This is the reason why these parameters were selected for biomolecule detection.

Two series of measurements were then performed: (1) estimation of the sensitivity of the target biomolecules detection (detecting model DNA oligonucleotides—synthetic analogues of miRNAs—in buffer solution); and (2) detection of miRNAs isolated from blood plasma samples of ovarian cancer patients.

### 3.2. Biospecific Detection of oDNAs—Synthetic Analogues of Target miRNAs

A SOI-NW chip sensitized with oDNA probes was used for highly sensitive detection of complementary oDNA targets (CS 1, CS 2, and CS 3) at ultra-low concentrations (within the concentration range from 1.1 × 10^−17^ M to 1.1 × 10^−14^ M) in buffer solution. Since the SOI-NW chip with n-type conductivity was employed, hybridization of oDNA targets with the oDNA probe corresponded to adsorption of negatively charged oDNA molecules on the nanowire surface, thus resulting in negative charge accumulation on this surface. Negative charge accumulation should reduce the nanowire conductivity [36,58], thus resulting in a decrease in the biosensor signal at a constant gate voltage *V_g_*. This is what was actually observed in our experiments.

**Figure 7:**
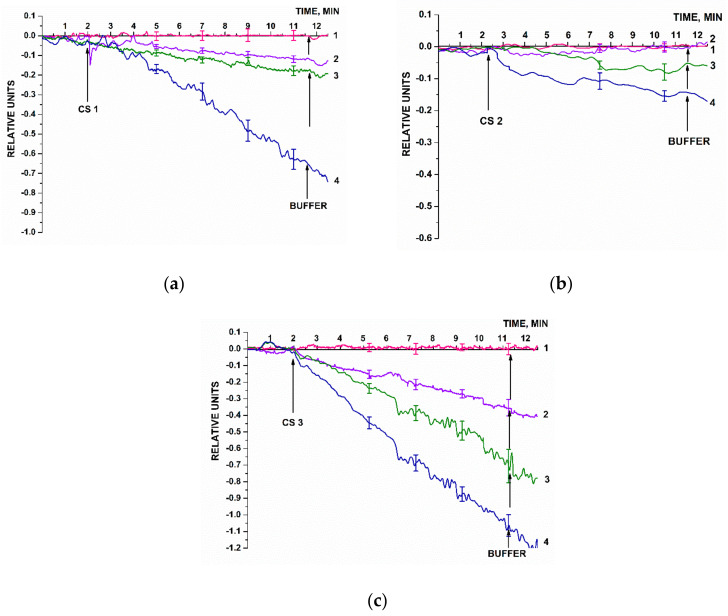
Typical sensogram curves recorded upon the biosensor-based detection of synthetic oDNA analogues of target miRNAs, oligonucleotides CS 1 (**a**), CS 2 (**b**), and or CS 3 (**c**) in buffer solutions. Experimental conditions: SOI-NW chip with n-type conductivity; nanowires were sensitized with oDNA probes (probe_1, probe_2, and probe_3); 1 mM PPMB, *V_g_* = 50 V, *V_ds_* = 0.2 V; solution volume in the measuring cell was 450 µL. Concentrations of the solutions of oDNA targets in the cell were as follows: 1.1 × 10^–17^ M (1, pink curve); 1.1 × 10^–16^ M (2, violet curve); 1.1 × 10^–15^ M (3, olive curve); and 1.1 × 10^–14^ M (4, blue curve). The number of technical replicates, *n* = 3. Arrows indicate the time point of addition of the solution of the oDNA target into the cell, and the washing with pure buffer.

Figure 7 displays typical sensorgram curves recorded in these experiments. Solutions of either CS 1, CS 2, or CS 3 were added to 1 mM PPMB in the measuring cell according to the procedure described in Section 2.8.

Figure 7 clearly demonstrates that the signal level predictably decreased after the analyzed CS solutions had been added (in all the cases except for the solution with target oDNA concentration of 10^−17^ M). That is, adsorption of negatively charged oDNA target molecules onto the nanowire surface was observed. Then, the amplitude of the decrease in the biosensor signal decreased with decreasing the concentration of oDNA target molecules. After the oDNA solution had been replaced with pure oDNA-free buffer, the signal level remained unchanged for a certain time. This can be explained by a very slow dissociation of the probe–target complexes formed on the nanowire surface. Therefore, when repeating the measurements, after washing with buffer, the cell was additionally washed with 50 mL of deionized water at 60 °C. After that, the initial signal level was restored, making it possible to use the chip multiple times.

In Figure 7, pink sensorgram curves indicate the signal obtained upon analysis of 1.1 × 10^−17^ M solutions of CS 1, CS 2, and CS 3 oligonucleotides. For all three oligonucleotides, these sensograms indicate no change in the signal level at such a low concentration of the target nucleotides. At a 10-times higher oDNA concentration (1.1 × 10^−16^ M, violet curve), a decrease in the signal from respective nanowires is observed. It is to be noted that for CS 2 oligonucleotide, however, the signal decreased distinctly only at much higher (1.1 × 10^−16^ and 1.1 × 10^−15^ M) target oDNA concentrations. The sensograms shown in Figure 7 indicate that within the concentration range studied, the higher the target oDNA concentration is, the greater the amplitude of the decrease in the biosensor signal.

Hence, the lowest concentration of the target oDNA detectable in the buffer solution using the SOI-NW biosensor was 1.1 × 10^−16^ M.

**Figure 8:**
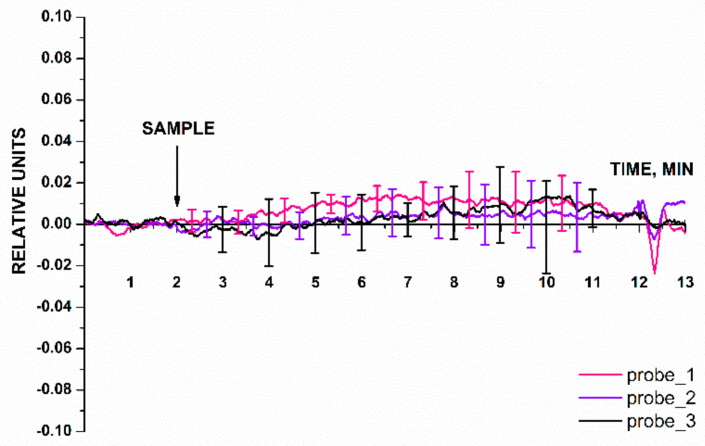
Typical sensorgram curves recorded in the blank experiments, in which the oDNA-free buffer was added into the measuring cell instead of target oDNA solution. Experimental conditions: nanowires with immobilized oDNA probes (probe_1, probe_2, and probe_3); 1 mM PPMB; *V_g_* = 50 V, *V_ds_* = 0,2 V; solution volume in the measuring cell was 450 µL. Number of technical replicates, *n* = 5. The arrow indicates the time point when the buffer under study (SAMPLE) was added.

The blank experiments were performed using an oDNA-free buffer instead of target oDNA solution (Figure 8). The pure buffer was added to the cell using the same procedure as that for the target oDNA solutions. Figure 8 displays the results of the blank experiments: signal dependences for the nanowires with immobilized probe_1, probe_2, and probe_3.

The data presented in Figure 8 indicate that the biosensor signal level at the time point when the buffer had been added into the measuring cell was comparable to that of the background signal. For the three working nanowires in the blank experiment, the response was ≤0.05 of the baseline signal. The results of the blank experiment demonstrate that the signal after buffer addition did not contribute to the signal recorded in the working experiment (in which the buffer containing the target oDNA molecules was used).

### 3.3. Biospecific Detection of miRNAs Isolated from Blood Plasma Samples

**Figure 9:**
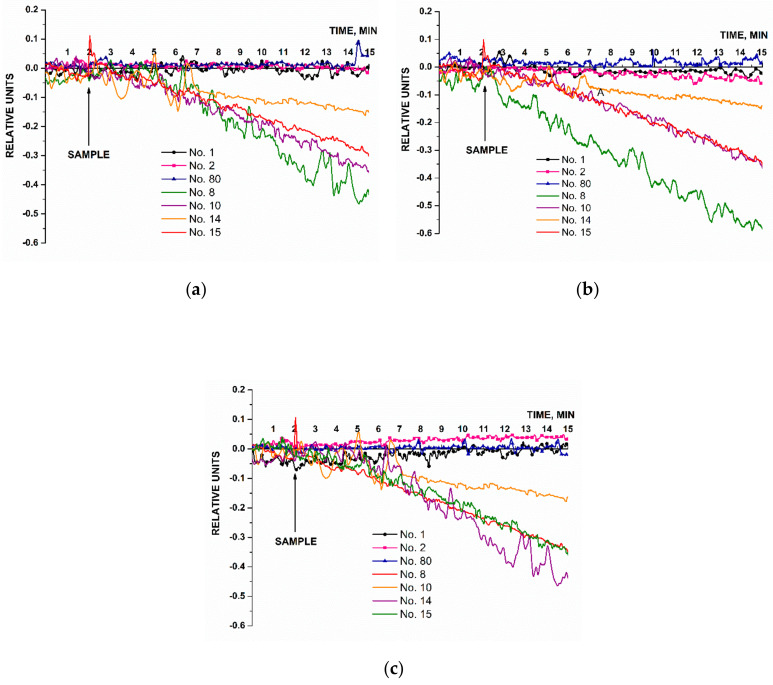
The results of biospecific detection of miRNAs isolated from blood plasma samples. Experimental conditions: SOI-NW chip with n-type conductivity; nanowires with immobilized oDNA probes: probe_1 (**a**), probe_2 (**b**), and probe_3 (**c**); 1 mM PPMB; *V_g_* = 50 V, *V_ds_* = 0.2 V; liquid volume in the measuring cell was 107 µL. Analyzed samples: samples No. 8, No. 10, No. 14, and No. 15 isolated from blood plasma of patients with ovarian cancer (olive, purple, orange, and red curves without markers); control sample No. 80 isolated from the plasma sample collected from a patient with breast cancer (blue curve, triangles); control samples No. 1 and No. 2 isolated from plasma samples collected from healthy volunteers (black curve, circles and pink curve, squares, respectively). The arrow indicates the time point when the analyzed solution containing isolated miRNAs was added.

The second series of experiments was performed in order to estimate whether or not it is possible to detect three types of miRNAs (miRNA-21, miRNA-141, and miRNA-200a) isolated from blood plasma samples of patients diagnosed with ovarian cancer. Figure 9 presents the results of sensing miRNAs isolated from blood plasma samples. Each panel in Figure 9 shows the examples of sensorgram curves recorded for one of the three working nanowires with immobilized probes: probe_1 (Figure 9a); probe_2 (Figure 9b); and probe_3 (Figure 9c). The group of sensorgram curves without markers presents the results of sensing miRNA samples isolated from blood plasma of ovarian cancer patients (four samples). The sensorgram curves with markers show the results obtained for the control samples: miRNAs isolated from blood plasma of healthy volunteers (two samples) and a patient with a verified diagnosis of breast cancer (one sample).

The results presented in Figure 9 demonstrate that the signal level changed considerably after solutions of miRNAs isolated from plasma of ovarian cancer patients had been added, similar to changes observed for the signals recorded in the experiments on the detection of the model oDNA targets (Figure 7). Upon analysis of the miRNA samples isolated from plasma samples No. 8, No. 10, No. 14, and No. 15, we observed that the levels of the signals from the nanowires sensitized with oDNA probe_1 (Figure 9a), probe_2 (Figure 9b), and probe_3 (Figure 9c) decreased. In the control experiments, a different trend was revealed for the signal obtained with solutions of miRNAs isolated from blood plasma samples of breast cancer patients (Figure 9a–c, blue curve) and plasma samples of healthy volunteers (Figure 9a–c, black and pink curves). Namely, the signal level either remained unchanged, or changed negligibly (the changes in the signal level were comparable with the background signal level). 

According to these findings, the miRNAs selected in this study as targets were successfully detected using the SOI-NW biosensor only in the samples collected from patients with a verified diagnosis of ovarian cancer, while not being detected in the control samples.

**Figure 10:**
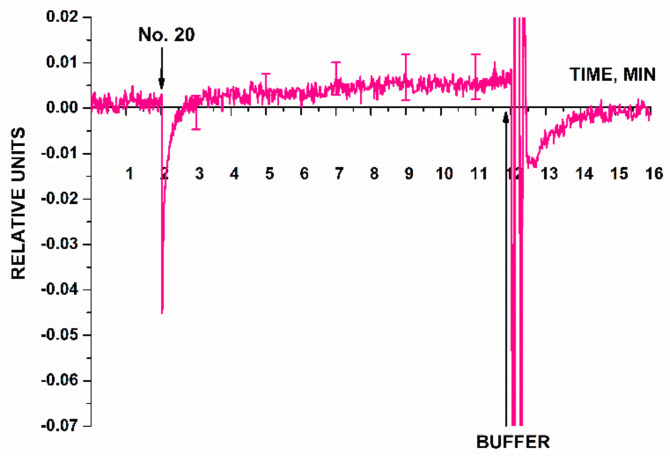
Typical sensogram obtained in the additional control experiment. The signal from the nanowire sensitized with immobilized oDNA probe not specific to any target miRNA (probe_5) was recorded in the case of adding miRNA solution isolated from the plasma sample of a patient with a verified diagnosis of ovarian cancer (No. 20). Experimental conditions: SOI-NW chip with n-type conductivity; the nanowire with immobilized oDNA probe (probe_5); 1 mM PPMB; pH 7.4; *V_g_* = 50 V, *V_ds_* = 0.4 V; the total solution volume in the cell was 107 µL. Number of technical replicates, n = 3. Arrows indicate the time points of addition of the analyzed miRNA solution and the pure washing buffer into the cell.

An additional control experiment was also performed in this experimental series using a nanowire with an immobilized oDNA probe, which was not specific to any cancer biomarker (probe_5). The solution of miRNAs isolated from plasma sample of a patient with ovarian cancer was analyzed. Figure 10 displays typical sensogram obtained in this additional control experiment.

According to the sensogram shown in Figure 10, this additional control experiment has shown that the signal from a nanowire sensitized with a probe non-specific to any of the selected target miRNAs remained virtually unchanged after the analyzed solution of the working sample had been added into the measuring cell. After washing with 1 mM PPMB, the signal intensity was restored to the baseline, thus indicating that adsorbed molecules had been removed from the nanowire surface. Therefore, the findings are indicative of biospecificity of the signal from the SOI-NW biosensor recorded in the experiments focusing on detection of target miRNAs (Figure 9). 

## 4. Discussion

This study was aimed at the development of a method of highly sensitive label-free detection of miRNAs (miRNA-21, miR-141, and miR-200a), which were reported to be associated with ovarian cancer [6,12]. At the first stage of our study, the sensitivity of the method developed was assessed in a series of experiments aiming to detect the model oDNA targets in buffer solutions with known concentrations of target molecules. The model oDNAs designated as CS in this study represented synthetic analogues of the target miRNAs (miRNA-21, miRNA-141, and miRNA-200a). The lowest concentration of these synthetic oDNA analogues, detectable with our SOI-NW biosensor in potassium phosphate buffer, was 1.1 × 10^−16^ M. This value is comparable to that obtained by Zhang et al. upon miRNA detection of with a nanowire biosensor sensitized with peptide nucleic acid (PNA) molecular probes [59]. These authors have reported that with such a biosensor, target miRNA molecules were detectable at 10^−15^ M concentration [59]. As discussed in Introduction, bioassay systems, which allow one to detect target biomolecules at 10^−13^ M and lower concentrations, are in great demand for early diagnosis of diseases in humans [11,22,46,60]. Therefore, the approach proposed herein is undoubtedly a highly sensitive method, since it has been demonstrated experimentally that biospecific detection of biomolecules within the subfemtomolar (10^−16^ M) concentration range is feasible.

In the second series of experiments, we demonstrated that the method developed can well be used to study biological samples. It was shown that it allows one to reliably detect miRNAs, isolated from plasma samples of ovarian cancer patients. The assay was shown to be biospecific: no biosensor signal was registered in the case of solutions containing miRNAs isolated from the plasma samples of either breast cancer patients or healthy volunteers.

This study shows that highly sensitive label-free detection of miRNAs can be performed using a SOI-NW biosensor. Undoubtedly, a large-scale study involving many samples from patients and healthy volunteers or patients with other gynecologic cancers needs to be performed in order to find out whether or not miRNAs selected in this study are biomarkers of ovarian cancer. The objective of our work was to demonstrate that it is fundamentally possible to detect miRNAs, associated with ovarian cancer as evidenced in the literature, using a novel nanotechnology-based approach [50,51,61]. The nearest rival of the proposed approach is real-time PCR, which allows one to detect low concentrations of target nucleic acids (typically ~ten DNA copies per reaction in 100 µL). However, PCR is extremely sensitive to sample contamination due to using the amplification reaction, which can lead to false results—in contrast to the non-amplification method proposed herein [16]. We believe that the fact that sample preparation for analysis involves neither labeling nor amplification and is extremely important for the development of novel diagnostic systems.

## 5. Conclusions

This study demonstrates that it is fundamentally possible to perform label-free sensing of miRNA-21, miRNA-141, and miRNA-200a isolated from plasma samples collected from patients diagnosed with ovarian cancer. The detection was performed using SOI-NW biosensor chips manufactured employing the CMOS-compatible technology intended for large-scale chip manufacturing; therefore, the application of the proposed method can potentially proceed from the laboratory-scale level to the level of routine bioassay. Due to the high concentration sensitivity of the assay (10^−16^ M), and the label-free procedure of miRNA sample preparation, there is no need to use the amplification reaction. The feasibility of real-time signal detection and multiplexed assay are the factors contributing to the advances in development of highly sensitive bioanalytical systems, based on SOI-NW molecular detectors, for the early revelation of ovarian cancer in women. 

## Figures and Tables

**Table 1:** Examples of biosensors employed for the detection of miRNAs, associated with ovarian cancer, and concentration detection limits attainable with these biosensors.

miRNA	Expression Pattern	Type of Tissue	Detection Limit	Biosensor Used	Reference
miRNA-21	Upregulated	Plasma, serum	0.32 × 10^−15^ M	Biosensor based on biomimetic fluorescence resonance energy transfer	[6,21,32]
miRNA-141	Upregulated	Plasma	0.94 × 10^−15^ M	Ratiometric electrochemical biosensor	[6,21,33]
miRNA-200a	Upregulated	Plasma	8.4 × 10^−15^ M	Electrochemical biosensor based on ZnS quantum dots	[6,34]
miRNA-155	Downregulated	Serum	5.5 × 10^−15^ M	Fluorescent biosensor based on “sandwich-type” hybridization of oligonucleotides	[6,21,35]

**Table 2:** Nucleotide sequences of oDNA probes immobilized on the nanowire surface.

Designation of oDNA Probes	Sequence of oDNA Probe
probe_1	5′- (NH_2_)-T_10_ TGTCAGACAGCCCATCGACTGGTGTTGCCATGAGATTCAA CAGTCAACATCAGTCTGATAAGCTACCCG
probe_2	5′- (NH_2_)-T_10_ GAACCCACCCGCGAGCCATCTTTACCAGACAGTGTTAGG AGCTTCACAATTAGACCATCCAACACTGTACTGGAAGATGGACCCAGGGCCGGCCG
probe_3	5′- (NH_2_)-T_10_ GCGGGTCACCTTTGAACATCGTTACCAGACAGTGTTAGA GTCAAGCTGGGAAATCCAGCACTGTCCGGTAAGATGCTCACAGGGGCCCG

**Table 4:** The characteristics of blood plasma samples.

	Sample	Age	Sex	Diagnosis	TNM Stage
Working samples	No. 8	49	female	left ovarian cancer	-
	No. 10	47	female	ovarian cancer	T3cN0M0
	No. 14	39	female	ovarian cancer	T3NxM0
	No. 15	68	female	ovarian Sertoli cell tumor	T3cNxM0
	No. 20	52	female	ovarian cancer	T1ANXM0
Control	No. 80	45	female	breast cancer	T2N0M0
	No. 1	53	female	healthy volunteer	-
	No. 2	18	female	healthy volunteer	-

## Data Availability

The data obtained throughout the experiments can be provided by Yu.D.I. upon reasonable request.

## References

[1] Kossaï M., Leary A., Scoazec J.-Y., Genestie C. (2018). Ovarian Cancer: A Heterogeneous Disease. Pathobiology.

[2] Penny S.M. (2020). Ovarian Cancer: An Overview. Radiol. Technol..

[3] Siegel R.L., Miller K.D., Fuchs H.E., Jemal A. (2022). Cancer Statistics, 2022. CA A Cancer J. Clin..

[4] Key Statistics for Ovarian Cancer. https://www.cancer.org/cancer/ovarian-cancer/about/key-statistics.html#written_by.

[5] Stewart C., Ralyea C., Lockwood S. (2019). Ovarian Cancer: An Integrated Review. Semin. Oncol. Nurs..

[6] Ghafouri-Fard S., Shoorei H., Taheri M. (2020). MiRNA Profile in Ovarian Cancer. Exp. Mol. Pathol..

[7] Bonifácio V.D.B., Serpa J. (2020). Ovarian Cancer Biomarkers: Moving Forward in Early Detection. Tumor Microenvironment. Advances in Experimental Medicine and Biology.

[8] Zhang M., Cheng S., Jin Y., Zhao Y., Wang Y. (2021). Roles of CA125 in Diagnosis, Prediction, and Oncogenesis of Ovarian Cancer. Biochim. Et Biophys. Acta BBA Rev. Cancer.

[9] Djoba Siawaya J.F., Roberts T., Babb C., Black G., Golakai H.J., Stanley K., Bapela N.B., Hoal E., Parida S., van Helden P. (2008). An Evaluation of Commercial Fluorescent Bead-Based Luminex Cytokine Assays. PLoS ONE.

[10] Chen S.-N., Chang R., Lin L.-T., Chern C.-U., Tsai H.-W., Wen Z.-H., Li Y.-H., Li C.-J., Tsui K.-H. (2019). MicroRNA in Ovarian Cancer: Biology, Pathogenesis, and Therapeutic Opportunities. Int. J. Environ. Res. Public Health.

[11] Elias K.M., Guo J., Bast R.C. (2018). Early Detection of Ovarian Cancer. Hematol. Oncol. Clin. N. Am..

[12] Garzon R., Croce C.M. (2011). MicroRNAs and Cancer: Introduction. Semin. Oncol..

[13] Deb B., Uddin A., Chakraborty S. (2018). MiRNAs and Ovarian Cancer: An Overview. J. Cell Physiol..

[14] Kilic T., Erdem A., Ozsoz M., Carrara S. (2018). MicroRNA Biosensors: Opportunities and Challenges among Conventional and Commercially Available Techniques. Biosens. Bioelectron..

[15] Tian T., Wang J., Zhou X. (2015). A Review: MicroRNA Detection Methods. Org. Biomol. Chem..

[16] Leonardi A.A., Lo Faro M.J., Irrera A. (2021). Biosensing Platforms Based on Silicon Nanostructures: A Critical Review. Anal. Chim. Acta.

[17] Várallyay É., Burgyán J., Havelda Z. (2008). MicroRNA Detection by Northern Blotting Using Locked Nucleic Acid Probes. Nat. Protoc..

[18] Beckers M., Mohorianu I., Stocks M., Applegate C., Dalmay T., Moulton V. (2017). Comprehensive Processing of High-Throughput Small RNA Sequencing Data Including Quality Checking, Normalization, and Differential Expression Analysis Using the UEA SRNA Workbench. RNA.

[19] Chen J., Zhou X., Ma Y., Lin X., Dai Z., Zou X. (2016). Asymmetric Exponential Amplification Reaction on a Toehold/Biotin Featured Template: An Ultrasensitive and Specific Strategy for Isothermal MicroRNAs Analysis. Nucleic Acids Res..

[20] Duan D., Zheng K., Shen Y., Cao R., Jiang L., Lu Z., Yan X., Li J. (2011). Label-Free High-Throughput MicroRNA Expression Profiling from Total RNA. Nucleic Acids Res..

[21] Shabaninejad Z., Yousefi F., Movahedpour A., Ghasemi Y., Dokanehiifard S., Rezaei S., Aryan R., Savardashtaki A., Mirzaei H. (2019). Electrochemical-Based Biosensors for MicroRNA Detection: Nanotechnology Comes into View. Anal. Biochem..

[22] Rissin D.M., Kan C.W., Campbell T.G., Howes S.C., Fournier D.R., Song L., Piech T., Patel P.P., Chang L., Rivnak A.J. (2010). Single-Molecule Enzyme-Linked Immunosorbent Assay Detects Serum Proteins at Subfemtomolar Concentrations. Nat. Biotechnol..

[23] Chung J.W., Kim S.D., Bernhardt R., Pyun J.C. (2005). Application of SPR biosensor for medical diagnostics of human hepatitis B virus (hHBV). Sens. Actuators B Chem..

[24] Rich R.L., Myszka D.G. (2004). Why you should be using more SPR biosensor technology. Drug Discov. Today Technol..

[25] Jang H.S., Park K.N., Kang C.D., Kim J.P., Sim S.J., Lee K.S. (2009). Optical fiber SPR biosensor with sandwich assay for the detection of prostate specific antigen. Opt. Commun..

[26] Singh P. (2016). SPR Biosensors: Historical Perspectives and Current Challenges. Sens. Actuators B Chem..

[27] Ivanov A.S., Medvedev A., Ershov P., Molnar A., Mezentsev Y., Yablokov E., Kaluzhsky L., Gnedenko O., Buneeva O., Haidukevich I. (2014). Protein interactomics based on direct molecular fishing on paramagnetic particles: Practical realization and further SPR validation. Proteomics.

[28] Florinskaya A., Ershov P., Mezentsev Y., Kaluzhskiy L., Yablokov E., Medvedev A., Ivanov A. (2018). SPR Biosensors in Direct Molecular Fishing: Implications for Protein Interactomics. Sensors.

[29] Jing J.-Y., Wang Q., Zhao W.-M., Wang B.-T. (2019). Long-range surface plasmon resonance and its sensing applications: A review. Opt. Lasers Eng..

[30] Liu Y.-Q., Kong L.-B., Liu P.-K. (2016). Long-range spoof surface plasmons on the doubly corrugated metal surfaces. Optics Commun..

[31] Awang M.S., Bustami Y., Hamzah H.H., Zambry N.S., Najib M.A., Khalid M.F., Aziah I., Abd Manaf A. (2021). Advancement in Salmonella Detection Methods: From Conventional to Electrochemical-Based Sensing Detection. Biosensors.

[32] Sun Y., Yao Y., Wang B., Li Y., Li S., Sui Y., Qiu B. (2021). Study on the Biosensor Based on Biomimetic PDA Vesicles Fluorescence Resonance Energy Transfer for the Determination of Ovarian Cancer Marker MiRNA-21. Anal. Sci..

[33] Khodadoust A., Nasirizadeh N., Taheri R.A., Dehghani M., Ghanei M., Bagheri H. (2022). A Ratiometric Electrochemical DNA-Biosensor for Detection of MiR-141. Microchim. Acta.

[34] Moazampour M., Zare H.R., Shekari Z. (2021). Femtomolar Determination of an Ovarian Cancer Biomarker (MiR-200a) in Blood Plasma Using a Label Free Electrochemical Biosensor Based on l-Cysteine Functionalized ZnS Quantum Dots. Anal. Methods.

[35] Afzalinia A., Mirzaee M. (2020). Ultrasensitive Fluorescent MiRNA Biosensor Based on a “Sandwich” Oligonucleotide Hybridization and Fluorescence Resonance Energy Transfer Process Using an Ln(III)-MOF and Ag Nanoparticles for Early Cancer Diagnosis: Application of Central Composite Design. ACS Appl. Mater. Interfaces.

[36] Ambhorkar P., Wang Z., Ko H., Lee S., Koo K., Kim K., Cho D. (2018). Nanowire-Based Biosensors: From Growth to Applications. Micromachines.

[37] Zhang G.-J., Ning Y. (2012). Silicon Nanowire Biosensor and Its Applications in Disease Diagnostics: A Review. Anal. Chim. Acta.

[38] Ivanov Y.D., Malsagova K.A., Goldaeva K.V., Pleshakova T.O., Shumov I.D., Galiullin R.A., Kapustina S.I., Iourov I.Y., Vorsanova S.G., Ryabtsev S.V. (2022). “Silicon-On-Insulator”-Based Nanosensor for the Revelation of MicroRNA Markers of Autism. Genes.

[39] Ivanov Y.D., Goldaeva K.V., Malsagova K.A., Pleshakova T.O., Galiullin R.A., Popov V.P., Kushlinskii N.E., Alferov A.A., Enikeev D.V., Potoldykova N.V. (2021). Nanoribbon Biosensor in the Detection of MiRNAs Associated with Colorectal Cancer. Micromachines.

[40] Malsagova K.A., Pleshakova T.O., Kozlov A.F., Galiullin R.A., Popov V.P., Tikhonenko F.V., Glukhov A.V., Ziborov V.S., Shumov I.D., Petrov O.F. (2021). Detection of Influenza Virus Using a SOI-Nanoribbon Chip, Based on an N-Type Field-Effect Transistor. Biosensors.

[41] Malsagova K.A., Pleshakova T.O., Galiullin R.A., Kozlov A.F., Shumov I.D., Popov V.P., Tikhonenko F.V., Glukhov A.V., Ziborov V.S., Petrov O.F. (2020). Highly Sensitive Detection of CA 125 Protein with the Use of an N-Type Nanowire Biosensor. Biosensors.

[42] Ivanov Y.D., Malsagova K.A., Popov V.P., Pleshakova T.O., Kozlov A.F., Galiullin R.A., Shumov I.D., Kapustina S.I., Tikhonenko F.V., Ziborov V.S. (2021). Nanoribbon-Based Electronic Detection of a Glioma-Associated Circular MiRNA. Biosensors.

[43] Ivanov Y.D., Malsagova K.A., Pleshakova T.O., Galiullin R.A., Kozlov A.F., Shumov I.D., Popov V.P., Kapustina S.I., Ivanova I.A., Isaeva A.I. (2021). Aptamer-Sensitized Nanoribbon Biosensor for Ovarian Cancer Marker Detection in Plasma. Chemosensors.

[44] Condrat C.E., Thompson D.C., Barbu M.G., Bugnar O.L., Boboc A., Cretoiu D., Suciu N., Cretoiu S.M., Voinea S.C. (2020). MiRNAs as Biomarkers in Disease: Latest Findings Regarding Their Role in Diagnosis and Prognosis. Cells.

[45] Wang K., Yuan Y., Cho J.-H., McClarty S., Baxter D., Galas D.J. (2012). Comparing the MicroRNA Spectrum between Serum and Plasma. PLoS ONE.

[46] Patolsky F., Zheng G., Hayden O., Lakadamyali M., Zhuang X., Lieber C.M. (2004). Electrical Detection of Single Viruses. Proc. Natl. Acad. Sci. USA.

[47] Afrough B., Eakins J., Durley-White S., Dowall S., Findlay-Wilson S., Graham V., Lewandowski K., Carter D.P., Hewson R. (2020). X-ray Inactivation of RNA Viruses without Loss of Biological Characteristics. Sci. Rep..

[48] Abolaban F.A., Djouider F.M. (2021). Gamma Irradiation-Mediated Inactivation of Enveloped Viruses with Conservation of Genome Integrity: Potential Application for SARS-CoV-2 Inactivated Vaccine Development. Open Life Sci..

[49] Han S., Zou H., Lee J.-W., Han J., Kim H.C., Cheol J.J., Kim L.-S., Kim H. (2019). MiR-1307-3p Stimulates Breast Cancer Development and Progression by Targeting SMYD4. J. Cancer.

[50] Xu Y.-Z., Xi Q.-H., Ge W.-L., Zhang X.-Q. (2013). Identification of Serum MicroRNA-21 as a Biomarker for Early Detection and Prognosis in Human Epithelial Ovarian Cancer. Asian Pac. J. Cancer Prev..

[51] Gao Y., Wu J. (2015). MicroRNA-200c and MicroRNA-141 as Potential Diagnostic and Prognostic Biomarkers for Ovarian Cancer. Tumor Biol..

[52] Langhe R., Norris L., Saadeh F.A., Blackshields G., Varley R., Harrison A., Gleeson N., Spillane C., Martin C., O’Donnell D.M. (2015). A Novel Serum MicroRNA Panel to Discriminate Benign from Malignant Ovarian Disease. Cancer Lett..

[53] Zuberi M., Mir R., Das J., Ahmad I., Javid J., Yadav P., Masroor M., Ahmad S., Ray P.C., Saxena A. (2015). Expression of Serum MiR-200a, MiR-200b, and MiR-200c as Candidate Biomarkers in Epithelial Ovarian Cancer and Their Association with Clinicopathological Features. Clin. Transl. Oncol..

[54] ExtractRNA A Reagent for the Isolation of Total RNA from Biological Samples. Catalog Number BC032. Instructions for Use. https://evrogen.ru/kit-user-manuals/extractRNA.pdf.

[55] Mattson G., Conklin E., Desai S., Nielander G., Savage M.D., Morgensen S. (1993). A Practical Approach to Crosslinking. Mol. Biol. Rep..

[56] Stern E., Wagner R., Sigworth F.J., Breaker R., Fahmy T.M., Reed M.A. (2007). Importance of the Debye Screening Length on Nanowire Field Effect Transistor Sensors. Nano Lett..

[57] Purwidyantri A., Domingues T., Borme J., Guerreiro J.R., Ipatov A., Abreu C.M., Martins M., Alpuim P., Prado M. (2021). Influence of the Electrolyte Salt Concentration on DNA Detection with Graphene Transistors. Biosensors.

[58] Shen M.-Y., Li B.-R., Li Y.-K. (2014). Silicon Nanowire Field-Effect-Transistor Based Biosensors: From Sensitive to Ultra-Sensitive. Biosens. Bioelectron..

[59] Zhang G.-J., Chua J.H., Chee R.-E., Agarwal A., Wong S.M. (2009). Label-free direct detection of MiRNAs with silicon nanowire biosensors. Biosens. Bioelectron..

[60] Zhu Z., Chen Z., Wang M., Zhang M., Chen Y., Yang X., Zhou C., Liu Y., Hong L., Zhang L. (2022). Detection of Plasma Exosomal MiRNA-205 as a Biomarker for Early Diagnosis and an Adjuvant Indicator of Ovarian Cancer Staging. J. Ovarian. Res..

[61] Wu L., Qu X. (2015). Cancer Biomarker Detection: Recent Achievements and Challenges. Chem. Soc. Rev..

